# Sol-Gel Processed Cobalt-Doped Methylated Silica Membranes Calcined under N_2_ Atmosphere: Microstructure and Hydrogen Perm-Selectivity

**DOI:** 10.3390/ma14154188

**Published:** 2021-07-27

**Authors:** Lunwei Wang, Jing Yang, Ruihua Mu, Yingming Guo, Haiyun Hou

**Affiliations:** 1 School of Urban Planning and Municipal Engineering, Xi’an Polytechnic University, Xi’an 710048, China; wlw10212021@163.com (L.W.); yingming128@126.com (Y.G.); 2 School of Environment & Chemical Engineering, Xi’an Polytechnic University, Xi’an 710048, China; 20131102@xpu.edu.cn (R.M.); houhaiyun77@126.com (H.H.)

**Keywords:** cobalt-doped, hydrothermal stability, activation energy, H_2_ perm-selectivities, regenerated

## Abstract

Methyl-modified, cobalt-doped silica (Co/MSiO_2_) materials were synthesized by sol-gel technique calcined in N_2_ atmospheres, and membranes were made thereof by coating method. The effects of Co/Si molar ratio (*n*_Co_) on the physical-chemical constructions of Co/MSiO_2_ materials and microstructures of Co/MSiO_2_ membranes were systematically investigated. The gas permeance performance and hydrothermal stability of Co/MSiO_2_ membranes were also tested. The results show that the cobalt element in Co/MSiO_2_ material calcined at 400 °C exists not only as Si–O–Co bond but also as Co_3_O_4_ and CoO crystals. The introduction of metallic cobalt and methyl can enlarge the total pore volume and average pore size of the SiO_2_ membrane. The activation energy (*E*_a_) values of H_2_, CO_2_, and N_2_ for Co/MSiO_2_ membranes are less than those for MSiO_2_ membranes. When operating at a pressure difference of 0.2 MPa and 200 °C compared with MSiO_2_ membrane, the permeances of H_2_, CO_2_, and N_2_ for Co/MSiO_2_ membrane with *n*_Co_ = 0.08 increased by 1.17, 0.70, and 0.83 times, respectively, and the perm-selectivities of H_2_/CO_2_ and H_2_/N_2_ increased by 27.66% and 18.53%, respectively. After being steamed and thermally regenerated, the change of H_2_ permeance and H_2_ perm-selectivities for Co/MSiO_2_ membrane is much smaller than those for MSiO_2_ membrane.

## 1. Introduction

Hydrogen has been recognized as an ideal energy carrier because of its clean, renewable, and high-calorific value features [1]. However, the industrially produced hydrogen from water-gas conversion process or steam reforming process [2,3] contains some other contaminants (impurities), such as CO_2_, N_2_, CH_4_, CO, H_2_O, etc. Additionally, from an environmental point of view, refining and extracting hydrogen from industrial waste gas is necessary [4]. Therefore, in order to obtain high-purity hydrogen, it is necessary to separate H_2_ from the gas mixture. Consequently, the upgrading of hydrogen is of significant attention due to the versatile requirement for hydrogen with good purity in transportation, distributed heat, power generation, and other advanced applications [5]. Membrane separation technology has become an interesting alternative for separation and purification of hydrogen in the industry due to its energy-saving and excessive efficiency. Currently, the membranes for separating hydrogen from gas mixture mainly include inorganic membranes, such as palladium metal membranes, carbon molecular sieve membranes, and microporous ceramic membranes [6,7,8]. Among of them, microporous silica membranes are the most widely investigated because of their good chemical stability, large gas-permeation flux, and high selectivity.

However, pure silica membranes exhibit poor hydrothermal stability in high temperature and humid air. Because Si–O–Si linkages are damaged upon interplay with water, Si–OH hydroxyl agencies are formed, which creates destruction and reconstruction of Si–O–Si bonds in the silica structure, resulting in densification of the silica structure [9,10]. This has been identified as a drawback in the development of silica membranes for gas separation in moist environments for practical industrial applications. An extremely good quantity of work has been done to develop and improve the stability of silica membranes under hydrothermal conditions. For one thing, the incorporation of hydrophobic groups can improve the stability of silica membranes under hydrothermal conditions, such as octyl [11], phenyl [12], alkylamine [13], methyl [14,15], perfluorodecyl [16], etc., which effectively reduce the Si–OH concentration on the surface of silica membrane materials, thereby reducing the physical adsorption of water molecules and enhancing the hydrophobicity of silica membranes. For another, further improvement of hydrothermal stability of silica membranes have been researched by introducing different types of inorganic metal/metal oxides, such as aluminum [17], zirconium [18], nickel [19,20], titanium [21], cobalt [22,23], palladium [24,25], magnesium [26], niobium [27], PdCo [28], FeCo [29], etc. They were all added in the process of sol synthesis, which produced great beneficial effects. A large number of studies have found that by introducing metal, the structure of the membrane appears to be denser, and the structural stability of the membrane material is particularly improved. This is because the mixed oxide network structure formed by incorporating transition metals is more stable than amorphous silica materials [27,30]. Among them, cobalt (Co) is an excellent dopant, and the doping of Co to SiO_2_ matrix can reduce water adsorption, providing greater resistance by way of reducing the hydrophilicity of silica [31].

Numerous investigations have been conducted on silica materials/membranes modified by cobalt. For example, Smart et al. [32] synthesized methyl-modified Co/SiO_2_ membranes calcined at 630 °C under air atmosphere using methyltriethoxysilane (MTES) and tetraethylorthosilicate (TEOS) as the silica source and Co(NO_3_)_2_·6H_2_O as the cobalt source. When testing the permeability of single gas at 600 °C and a feed pressure of 600 KPa, it was observed that the H_2_ permeation reached 1.9 × 10^−7^ mol·m^−2^·Pa^−1^·s^−1^, and a H_2_/CO_2_ perm-selectivity exceeded 1500. Liu et al. [33] investigated the hydrothermal stability of the Co/SiO_2_ xerogels calcined at 630 °C in an air atmosphere under various hydrothermal treatment conditions. For unstable xerogels (cobalt/silicon < 0.25), their stability was significantly reduced due to steam content and exposure time, leading to a surface-area reduction of nearly 90%. However, it is found that the xerogels with high cobalt content (cobalt/silicon ≥ 0.25) contained Co_3_O_4_ and were more stable, with a surface-area reduction of less than 25%. Esposito et al. [34] prepared cobalt-doped silica nanocomposites with various cobalt contents (cobalt/silicon = 0.111, 0.250, and 0.428) by the sol-gel process. After treatment at 400 °C under air atmosphere, the lowest cobalt-loading, cobalt-doped silica nanocomposites appeared amorphous and contained solely tetrahedral complexes of Co^2+^, whilst Co_3_O_4_ was current as the solely crystalline section at greater cobalt content, besides the strong interaction of Co^2+^ ions with the siloxane matrix. Many research groups found that Co_3_O_4_ was the main existing form of cobalt in the Co/SiO_2_ material calcined under air atmosphere. However, Co_3_O_4_ is unstable in a hydrogen atmosphere and is easily reduced. According to Uhlmann et al. [35], after hydrogen reduction, the cobalt-doped xerogels calcined at 500 °C lost their crystal structure and showed no Co_3_O_4_ or CoO peaks but only a wide peak similar to that of amorphous silica. There have been a number of works revealing the effects of preparation conditions on the properties of cobalt-doped silica materials/membranes. Unfortunately, as far as we are aware, the influence of calcination atmospheres on the microstructures and characteristics of permeability for Co/SiO_2_ membrane is crucial, but it has rarely been reported before, especially under a non-oxidizing atmosphere, such as N_2_ atmosphere. Besides, there are few papers elaborating the influence of Co/Si molar ratio and methyl modification on the microstructures and characteristics of permeability for Co/SiO_2_ membrane.

In this work, methyl-modified Co/SiO_2_ (Co/MSiO_2_) materials and membranes with different Co/Si molar ratio (*n*_Co_) were prepared. The effect of *n*_Co_ on the physical-chemical structures and microstructures of Co/MSiO_2_ membrane calcined under N_2_ atmosphere was studied in detail. Characterization and results were attained by X-ray diffraction (XRD), X-ray photoelectron spectroscopy (XPS), Fourier transform infrared (FTIR) spectroscopy, N_2_ sorption/desorption measurements, transmission electron microscope (TEM), and scanning electron microscope (SEM). Some gas-permeation measurements of Co/MSiO_2_ membranes were performed and compared with each other. By observing the changes of gas-permeation characteristics before and after exposure to saturated steam, the hydrothermal stability of the Co/MSiO_2_ membranes was additionally investigated. Subsequently, regeneration performance of the Co/MSiO_2_ membranes for single gas was discussed.

## 2. Experimental Part

### 2.1. Fabrication of Methyl-Modified Co/SiO_2_ Sols

The methyl-modified Co/SiO_2_ (Co/MSiO_2_) sols were prepared via sol-gel process, using methyltriethoxysilane (MTES, purity 99%) and tetraethylorthosilicate (TEOS, p.a. grade) as silica sources, the cobaltous nitrate hexahydrate (Co(NO_3_)_2_·6H_2_O, purity 98%) as a cobalt source, and the nitric acid (HNO_3_, p.a. grade) as a catalyst in conjunction with absolute ethanol (EtOH, purity 99.7%) as a solvent. The specific synthesis process is as follows: in accordance to the molar ratio of MTES:TEOS:Co(NO_3_)_2_·6H_2_O:HNO_3_:EtOH:H_2_O = 0.8:1:*n*_Co_:0.085:8.5:6.8, the required quantity of MTES, TEOS, and Co(NO_3_)_2_·6H_2_O solution was completely dissolved in ethanol. Then, the mixed solution was placed in an ice-water bath and stirred intensely to make it fully mixed into a homogeneous solution. After the mixture solution of H_2_O and HNO_3_ was poured dropwise, the reaction mixture was then stirred continuously and refluxed in a water bath at 60 °C for 180 min. Therefore, a final Co/MSiO_2_ sol was obtained. The *n*_Co_ is Co/Si molar ratio, which is 0, 0.08, 0.15, and 0.5, respectively.

### 2.2. Fabrication of Unsupported Co/MSiO_2_ Materials

The prepared Co/MSiO_2_ sols were dried at 40 °C in a vacuum oven to prepare the dry gels. The obtained dry gels were then ground into fine powders and calcined under N_2_ atmosphere at 400 °C in a temperature-controlled tubular furnace with the temperature rising rate of 1 °C·min^−1^ for a resident time of 2 h to prepare the unsupported Co/MSiO_2_ materials.

### 2.3. Fabrication of Supported Co/MSiO_2_ Membranes

To obtain the supported Co/MSiO_2_ membranes, part of the above Co/MSiO_2_ sols was applied to the surface of porous α-Al_2_O_3_ composite discs (Hefei Shijie Membrane Engineering Co. Ltd., Hefei, China) by dip-coating method. The discs had a thickness of 4 mm, a diameter of 20 mm, a mean pore diameter of 100 nm, and a porosity of 40%. The dipping time was 6 s. After dipping, they were dried for 3 h at 40 °C in an electric heating blast drying oven and then calcined at 400 °C under N_2_ atmosphere in a tubular furnace for a resident time of 2 h. The process for dipping, drying, and calcination was performed once more in order to minimize any defects that might be occurred in the Co/MSiO_2_ membrane layer. The Co/MSiO_2_ membrane with *n*_Co_ = 0 is also referred to as MSiO_2_ membrane. The prepared supported MSiO_2_ and Co/MSiO_2_ membranes were used to test the permeances of H_2_, CO_2_, and N_2_.

### 2.4. Characterization

The material phase structure was determined by a Rigaku D/max-2550pc X-ray diffractometer (XRD, Rigaku D/max-2550pc, Hitachi, Tokyo, Japan) with CuKα radiation under the conditions of 40 kV and 40 mA. The functional groups of samples were characterized by Fourier transform infrared spectroscopy (FTIR, Nicolet 5700, Thermo Nicolet Corporation, WI, USA), and the wavelength measurement range was 400~4000 cm^−1^ by KBr compression method. The chemical components of Co/MSiO_2_ samples were performed by an X-ray photoelectron spectrometer (XPS, ESCALAB250xi, Thermo Scientific, MA, USA) with AlKα excitation. The transmission electron microscopy (TEM, JEM 2100F, JEOL, Tokyo, Japan) was used to analyse the crystallization of the Co/MSiO_2_ powders. The morphologies of surface and cross-sections for the membranes were observed by a scanning electron microscopy (SEM, JEOL JSM-6300, Hitachi, Tokyo, Japan) under 5 kV acceleration voltage. The BET surface area and pore volume of the samples was measured by N_2_ sorption/desorption isotherm with a specific surface area and pore-size analyzer (ASAP 2020, Micromeritics, GA, USA).

**Figure 1:**
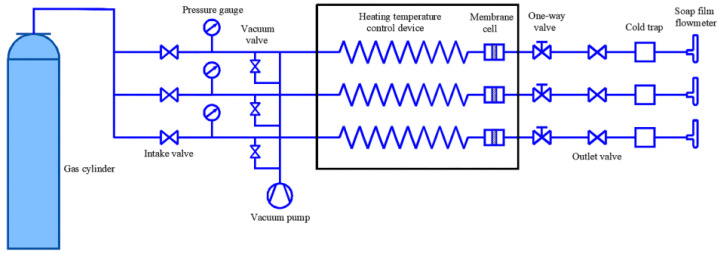
Schematic diagram of experimental devices for permeation measurement of single gas.

The schematic diagram of experimental devices used to test single gas-permeation measurement is shown in Figure 1. Before testing the experiments, pressure and temperature were kept at 0.35 MPa and 200 °C for 0.5 h until gas permeation achieved a stable state. The MSiO_2_ and Co/MSiO_2_ membranes were tested using high-purity H_2_, CO_2_, and N_2_, respectively. Additionally, the steam stability of MSiO_2_ and Co/MSiO_2_ membranes was examined by exposure to saturated steam at 200 °C for 10 days. After steam-stability test, the thermal regeneration of MSiO_2_ and Co/MSiO_2_ membranes was carried out at 350 °C with the aid of the equal calcination process as described above. The gas permeance was calculated as the usage of the outlet gas flow rate. The values of gas perm-selectivity were calculated from the ratio of individual gas-permeance values at the same transmembrane pressure difference and temperature.

## 3. Results

### 3.1. Phase Structure Analysis

**Figure 2:**
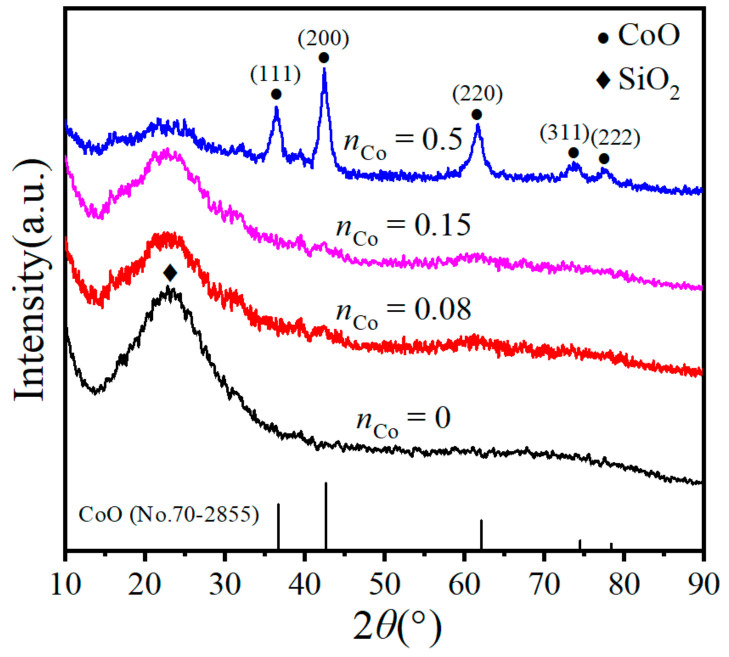
XRD patterns of unsupported Co/MSiO_2_ materials with various *n*_Co_ calcined at 400 °C under N_2_ atmosphere.

The XRD patterns of the unsupported Co/MSiO_2_ materials with various *n*_Co_ calcined at 400 °C under N_2_ atmosphere are provided in Figure 2. A distinct diffraction peak at about 2*θ* = 23° is assigned to the feature of amorphous SiO_2_ for all samples, and the peak intensity decreases with increasing *n*_Co_. It is probably because the introduction of cobalt atoms replaces the original silicon atoms and forms the Si–O–Co bonds, resulting ultimately in an increase in Si–O–Co bonds and a decrease in SiO_2_. When *n*_Co_ is equal to 0.5, significant absorption peaks appeared at 2*θ* = 36.57, 42.49, 61.64, 73.86, and 77.74°, which corresponds to the plane reflections of (111), (200), (220), (311), and (222) of CoO crystalline phase (PDF No. 70-2855), respectively, which indicates part of the cobalt is dispersed on the surface of the material in the shape of CoO. There are no peaks of CoO and Co_3_O_4_ in unsupported Co/MSiO_2_ material with *n*_Co_ = 0. However, the characteristic peak of CoO in the samples with *n*_Co_ = 0.08 and 0.15 is not obviously observed, which may be due to the fact that the CoO amount is too low or the size of the formed CoO is too small to be detected [36,37]. According to the literature, the XRD patterns of Co-doped silica powders with a cobalt mole fraction of 33% sintered at 550 °C with an existing Co_3_O_4_ peak [38]. In this paper, when *n*_Co_ is equal to 0.08–0.5, no characteristic peak of Co_3_O_4_ is observed in the samples calcined at 400 °C, which does not mean that Co_3_O_4_ is not present in the sample. This may be owing to the fact that the content of Co_3_O_4_ is small and cannot be detected by XRD [36].

The full-width at half maxima of the characteristic reflection with the highest intensity (200) was used to calculate the mean crystallite size with the aid of making use of the Scherrer equation [39]:

(1) $$D=\frac{k\lambda }{\beta \cos\theta }$$

where *D* is the size of CoO crystallites, *k* is the constant value of Scherrer (0.89), *λ* is the wavelength of X-ray source (0.154 nm), *β* is the full width at half maximum intensity, and *θ* is the Bragg angle. Hence, the mean size of CoO crystals in the samples with *n*_Co_ = 0.5 is calculated as 3.8 nm.

**Figure 3:**
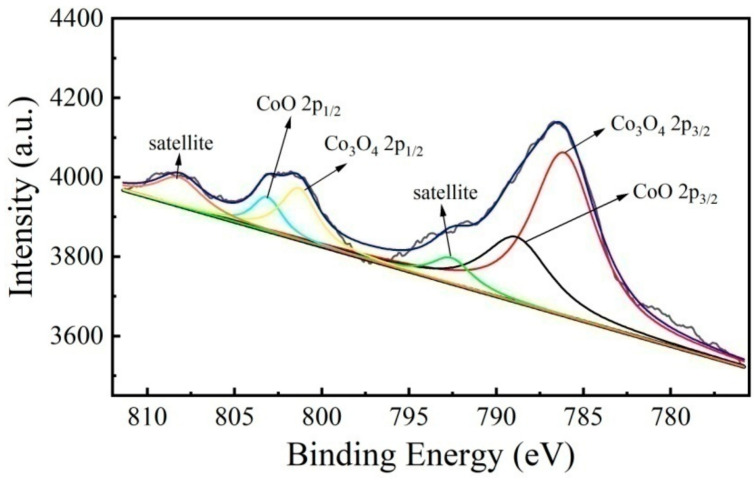
Co2p XPS spectra of unsupported Co/MSiO_2_ material with *n*_Co_ = 0.08 calcined at 400 °C under N_2_ atmosphere.

In order to further determine the presence of Co species in the unsupported Co/MSiO_2_ materials with *n*_Co_ = 0.08, 0.15, and 0.5 calcined at 400 °C under N_2_ atmosphere. We characterized the unsupported Co/MSiO_2_ material with *n*_Co_ = 0.08 by XPS. The XPS spectra curves for samples are displayed in Figure 3. The peaks at 786.2 eV and 801.3 eV are assigned to the 2p_3/2_ and 2p_1/2_ peaks of Co_3_O_4_, respectively, and the peaks of 789.1 eV and 803.3 eV correspond to the 2p_3/2_ and 2p_1/2_ peaks of CoO, respectively [40]. Additionally, two shake-up satellite peaks can be seen at 792.8 eV and 808.3 eV, which are because of the multi-electron excitation of Co^2+^ [28]. However, the binding energies of Co_3_O_4_ and CoO in the SiO_2_ material are higher than that of the pure component, as previously reported [41]. This is resulted from the formation of the Si–O–Co bond between Co oxides and Si atoms due to the interaction of electrons. Hence, the XPS investigation clearly indicated that the doped Co component is present in the oxide rather than the metal. This result was confirmed by XRD analysis. For the samples with even higher Co content, such as unsupported Co/MSiO_2_ materials with *n*_Co_ = 0.15 and 0. 5, the Co signal becomes very strong (especially the latter one), and it is obvious that the peaks of Co_3_O_4_ and CoO are found in the samples by spectrum analysis. Therefore, the results are not included for comparison.

### 3.2. FTIR Analysis

**Figure 4:**
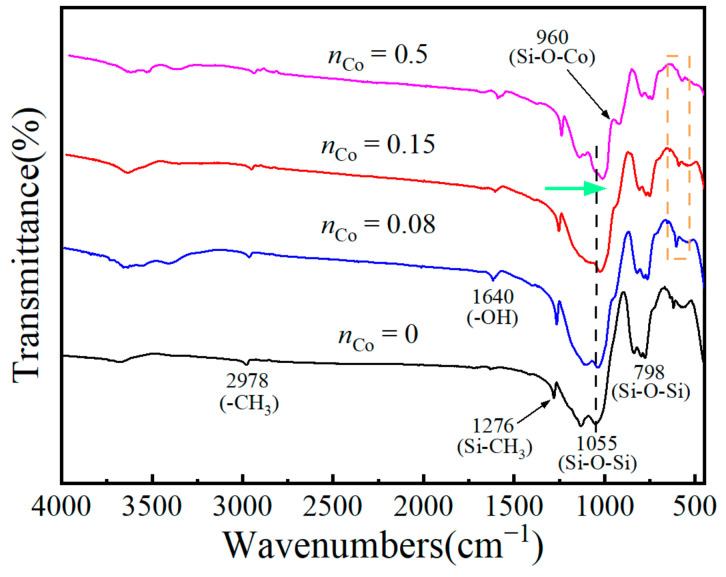
FTIR spectra curves of unsupported Co/MSiO_2_ materials with various *n*_Co_ calcined in N_2_ atmosphere at 400 °C (the green arrow represents the direction in which the Si−O−Si bonds centered at 1055 cm^−1^ moves; the dotted rectangle emphasizes the peaks at 571 and 664 cm^−1^).

To further consider the influence of *n*_Co_ on chemical structure for the unsupported Co/MSiO_2_ materials, the unsupported Co/MSiO_2_ materials with various *n*_Co_ calcined in N_2_ atmosphere at 400 °C are characterized by FTIR spectra, which are shown in Figure 4. In Figure 4, the peak appearing at about 2978 cm^−1^ is attributed to the –CH_3_ groups for TEOS and MTES. The absorption peak located at 1640 cm^−1^ is associated with the stretching and bending vibration of –OH groups [42] from the absorbed water and ethanol as well as Si–OH. Undoubtedly, the existence of 1276 cm^−1^ band indicates the stretching vibration of the Si–CH_3_ groups. The band located at 770–800 cm^−1^ is accompanied by a shoulder, which is attributed to the asymmetric tensile vibration of the Si–O–Si bonds [43]. For Co/MSiO_2_ material with *n*_Co_ = 0, the absorption peak at about 1055 cm^−1^ is also assigned to the vibration of Si–O–Si bonds. With the increasing *n*_Co_, the Si–O–Si bonds centered at 1055 cm^−1^ gradually shift to a lower value. The movement of Si–O–Si bonds indicates that Co enters the SiO_2_ lattice and that Si–O–Co bonds exist in the materials, which destroys the symmetry of SiO_2_ and causes the move of peak position. A similar phenomenon has been reported in other literature [44,45]. In addition, when *n*_Co_ = 0.5, an additional peak is found located at 960 cm^−1^ and corresponding to the Si–O–Co vibration, suggesting that cobalt enters into the silica framework and forms the Si–O–Co bonds. However, Si–O–Co bonds in the samples with *n*_Co_ = 0.08 and 0.15 are not obvious, which may be owing to the fact that the Si–O–Co bonds cannot be detected when the content of doped cobalt is small. Generally, the FTIR bands assigned to Co_3_O_4_ are located at 571 cm^−1^ and 664 cm^−1^ [46], but there is no obvious peak of Co_3_O_4_ in this figure, which does not imply that Co_3_O_4_ is not present in the samples. The reason may be that the content of Co_3_O_4_ is too small, and the peak is not revealed.

### 3.3. Pore-Structure Analysis

**Figure 5:**
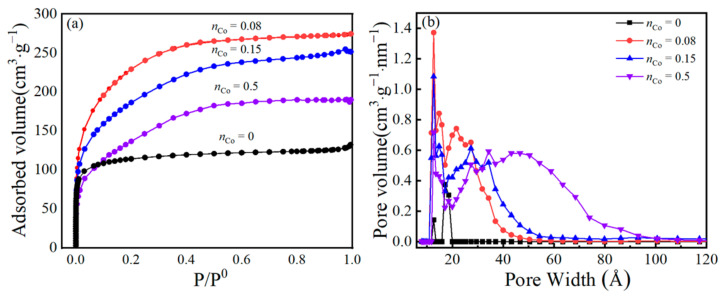
(**a**) N_2_ adsorption-desorption isotherms and (**b**) pore size distributions of unsupported Co/MSiO_2_ materials with various *n*_Co_ at 400 °C.

The physical characteristics of the prepared samples can be greatly influenced by their specific surface area and porous structure. The N_2_ adsorption-desorption isotherm curves of unsupported Co/MSiO_2_ materials with various *n*_Co_ at 400 °C are displayed in Figure 5a. As shown in Figure 5a, the isotherms for the four samples all show a similar trend, which can be categorized as type I isotherm. In the range of low relative pressure P/P_0_ < 0.1, a substantial amount of adsorption indicates that there is a giant quantity of micropores in the materials. As the relative pressure increases, the isotherm gradually increases, which confirms the existence of a small amount of mesopores. The N_2_ adsorption of unsupported Co/MSiO_2_ materials increases initially with the increasing *n*_Co_, then begins to decrease as *n*_Co_ > 0.08, which indicates the change of pore-volume variation trend. The distributions of pore size for all samples are depicted in Figure 5b. It is found that the unsupported Co/MSiO_2_ materials with *n*_Co_ = 0.08–0.5 have a wider pore size distribution and a bigger mean pore size than the sample with *n*_Co_ = 0. In addition, the pore diameters of all samples are mainly concentrated around 1.3 nm. The detailed information about the pore size and surface area for these four samples is provided in Table 1. It can be observed that, with the increases of *n*_Co_, the mean pore size increases, and the micropore volume decreases; the total pore volume and BET surface area increase until *n*_Co_ = 0.08, after which they begin to decrease. This is because the added cobalt atoms exist in the form of Si–O–Co bonds in the SiO_2_ skeleton, and the atomic radius of the cobalt atoms is larger than that of the silicon atoms, which plays a role in expanding the pores [45]. So, with the increase of *n*_Co_, the particle size, mean pore size, total pore volume, and surface area, the distribution of pore size becomes wider and shifts gradually to the direction of the mesopores. However, as *n*_Co_ > 0.08, in addition to the existence of cobalt in the skeleton in the amorphous form, there are also some cobalt oxides interspersed in the pores to block part of the pores, which leads to a decreasing in the pore volume and BET surface area. When *n*_Co_ = 0.08, the unsupported Co/MSiO_2_ material is more favorable for achieving a higher total pore volume (0.424 cm^3^·g^−1^) and BET surface area (775.344 m^2^·g^−1^), with the minimum mean pore diameter (2.34 nm). Therefore, the unsupported Co/MSiO_2_ material with *n*_Co_ = 0.08 is more favorable for gas separation.

### 3.4. TEM Analysis

**Figure 6:**
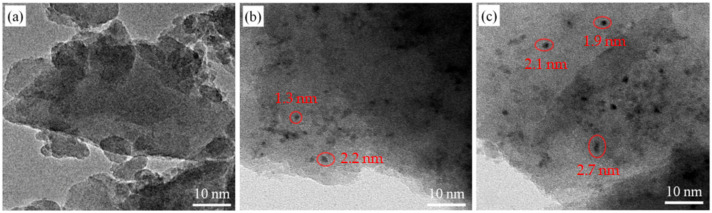
TEM images of unsupported Co/MSiO_2_ materials with *n*_Co_ = (**a**) 0, (**b**) 0.08, and (**c**) 0.15 calcined in N_2_ atmosphere at 400 °C.

To gain an insight into the Co species, the TEM analysis was performed to determine the presence of Co species in unsupported Co/MSiO_2_ materials with various *n*_Co_ calcined in N_2_ atmosphere at 400 °C. Figure 6 demonstrates the transmission electron microscope images for unsupported Co/MSiO_2_ materials with *n*_Co_ = 0, 0.08, and 0.15 calcined in N_2_ atmosphere at 400 °C. For these three samples, because of differences in electronic density, the darker-contrast particles can be attributed to cobalt oxide, while the lighter-contrast particles are attributed to silica carrier. In Figure 6a, amorphous silica can be observed on Co/MSiO_2_ material with *n*_Co_ = 0. In Figure 6b,c, a small amount of the CoO crystals are uniformly dispersed on the surface of Co/MSiO_2_ materials with *n*_Co_ = 0.08 and 0.15, and the particle size of the CoO crystals increases with the increasing *n*_Co_. Furthermore, the crystal size of CoO in Co/MSiO_2_ material with *n*_Co_ = 0.08 is in the range of 1.3–2.2 nm, and that in the Co/MSiO_2_ material with *n*_Co_ = 0.15 is in the range of 1.9–2.7 nm. Nevertheless, no Co_3_O_4_ particles were found on the outside surfaces of two samples, which does not imply that Co_3_O_4_ does not exist in the samples. This may be due to the tiny amount. For the unsupported Co/MSiO_2_ material with *n*_Co_ = 0.5, it is clear that particles of Co_3_O_4_ and CoO are scattered on the surface of the sample by TEM analysis because of a higher Co content. Thus, the sample is not introduced here. It can be seen that the conclusions obtained above are consistent with the results from the XRD and XPS analysis.

### 3.5. Gas-Permeance Analysis

**Figure 7:**
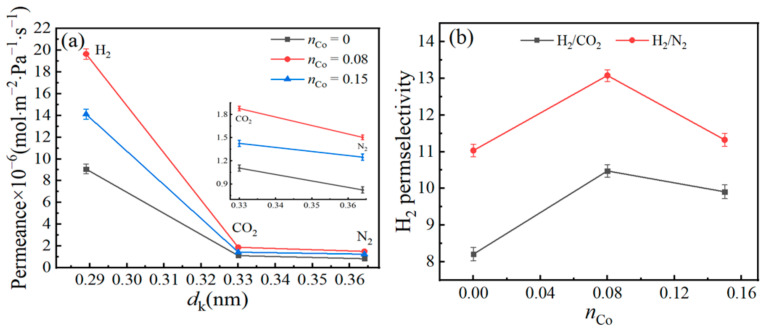
(**a**) Gases’ (H_2_, CO_2_, and N_2_) permeances versus gas molecules’ kinetic diameters (*d*_k_) and (**b**) H_2_ perm-selectivities of supported Co/MSiO_2_ membranes with various *n*_Co_ at a pressure difference of 0.2 MPa and 200 °C.

Based on the XRD, XPS, FTIR, N_2_ adsorption-desorption, and TEM results, the *n*_Co_ shows obvious impact on the physical-chemical structures of unsupported Co/MSiO_2_ materials. However, compared with the unsupported Co/MSiO_2_ materials with *n*_Co_ = 0.08 and 0.15, the pore volume and surface area for unsupported Co/MSiO_2_ materials with *n*_Co_ = 0.5 are smaller, whereas the mean pore size is larger. As far as we know, the permeation and selectivity of gas-separation membrane are dependent on the pore structure and surface area. It could suggest that the supported Co/MSiO_2_ membrane with *n*_Co_ = 0.5 is not suitable for gas-permeation experiments. Therefore, the supported Co/MSiO_2_ membrane with *n*_Co_ = 0.5 is not considered here. The permeances for gases (H_2_, CO_2_, and N_2_) to the gas molecules’ kinetic diameters (*d*_k_) and H_2_ perm-selectivities of Co/MSiO_2_ membrane with *n*_Co_ = 0, 0.08, and 0.15 at a pressure difference of 0.2 MPa and 200 °C are shown in Figure 7. The gas molecules’ kinetic diameters can be acquired from the report in [47]. The Co/MSiO_2_ membrane with *n*_Co_ = 0 is also referred to as MSiO_2_ membrane. From Figure 7a, as *n*_Co_ > 0.08, the permeances of Co/MSiO_2_ membrane to H_2_, CO_2_, and N_2_ appears to decrease. The H_2_ permeance of Co/MSiO_2_ membranes with *n*_Co_ = 0, 0.08, and 0.15 are 9.07 × 10^−6^, 1.97 × 10^−5^, and 1.41 × 10^−5^ mol·m^−2^·Pa^−1^·s^−1^, respectively. Compared with the Co/MSiO_2_ membrane with *n*_Co_ = 0, the H_2_, CO_2_, and N_2_ permeances of Co/MSiO_2_ membrane with *n*_Co_ = 0.08 increase by 1.17, 0.70, and 0.83 times, respectively. It can be seen from the pore structure analysis that the total pore volume and average pore diameters for the silica membranes enlarge slightly with increasing *n*_Co_, giving an explanation for the increase in gas permeance. Furthermore, for the same membrane, the order for permeance of gas molecules is N_2_ < CO_2_ < H_2_. The gas permeation decreases as the *d*_k_ increases, suggesting that all membranes show molecular sieve characteristics. These above results indicate that the porosity of the membrane shows a mean pore size of approximately 0.3 nm. It can be observed from Figure 7b that the perm-selectivities of H_2_/CO_2_ and H_2_/N_2_ for Co/MSiO_2_ membranes with various *n*_Co_ are all significantly higher than the ideal perm-selectivities of Knudsen diffusion, which are 4.69 and 3.74, respectively. Compared with MSiO_2_ membrane, the H_2_/CO_2_ and H_2_/N_2_ perm-selectivities of Co/MSiO_2_ membrane with *n*_Co_ = 0.08 increase by 27.66% and 18.53%, respectively. Therefore, the amplification of H_2_ perm-selectivities is not entirely ruled by the aid of molecular sieving; however, it may additionally be partly attributed to the improved adsorption of hydrogen by means of the Co/MSiO_2_ membrane matrix. The consequences of improved H_2_ perm-selectivities have additionally been mentioned for the Ni/SiO_2_ [20] and Pd/SiO_2_ [24] membranes, which have been ascribed to the enlarge affinity of H_2_ with the aid of the metallic particles. Moreover, when *n*_Co_ = 0.08, the H_2_ perm-selectivities of the Co/MSiO_2_ membrane reach the maximum value. However, as the *n*_Co_ continues to increase, the H_2_ perm-selectivities show a gradual decrease. The above consequences point out that the *n*_Co_ performs an advantageous function in the impact of gas permeation for the membrane, but it does not mean that the higher the *n*_Co_, the better the gas-permeation effect. Thus, the Co/MSiO_2_ membrane with *n*_Co_ = 0.08 has good gas permeability and selectivity, which is more appropriate for gas-permeation experiments.

**Figure 8:**
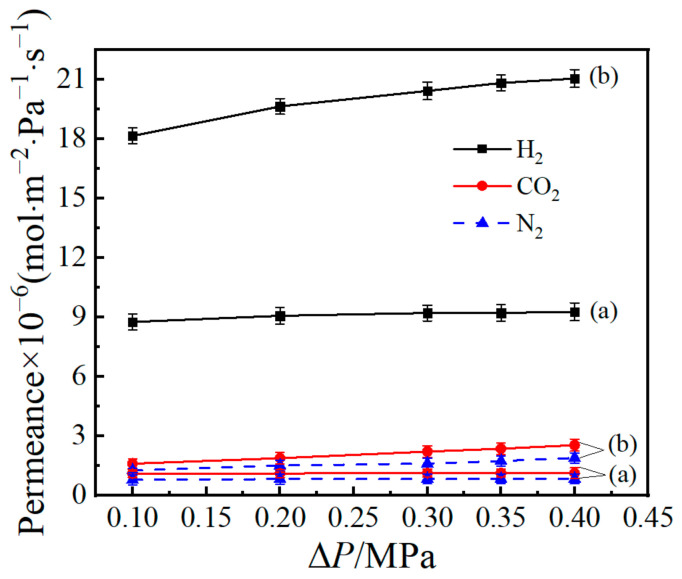
Influence of pressure difference on the gases’ (H_2_, CO_2_, and N_2_) permeances for supported (**a**) MSiO_2_ membrane and (**b**) supported Co/MSiO_2_ membrane with *n*_Co_ = 0.08 at 200 °C.

Figure 8 displays the influence for pressure difference on the gases’ (H_2_, CO_2_, and N_2_) permeances of MSiO_2_ membrane at 200 °C; their pressure difference is generally unbiased, whilst that for H_2_ in Co/MSiO_2_ membrane with *n*_Co_ = 0.08 increases significantly with growing pressure difference. It suggests that, due to the impact of the incorporated metal cobalt, the mechanism of H_2_ diffusion for Co/MSiO_2_ membrane is different from that for MSiO_2_ membrane. There are small mesopores on the membrane surface, which leads to the result that the obtained Co/MSiO_2_ membranes are accompanied by Knudsen diffusion. However, the doped metallic cobalt can improve the surface diffusion of H_2_ molecules in SiO_2_ membranes, and the growth of pressure is conducive to the adsorption of hydrogen. In addition, with the gradual increase of pressure, slight increases in the permeances of CO_2_ and N_2_ for Co/MSiO_2_ membrane are observed, which is due to the small influence of pressure on Knudsen diffusion, as previously reported in other literature [48,49]. Accordingly, the permeances of CO_2_ and N_2_ in Co/MSiO_2_ membrane are slightly dependent on pressure, whereas the H_2_ permeance increases within the pressure range due to the enhanced surface diffusion of hydrogen molecules by the cobalt particles.

**Figure 9:**
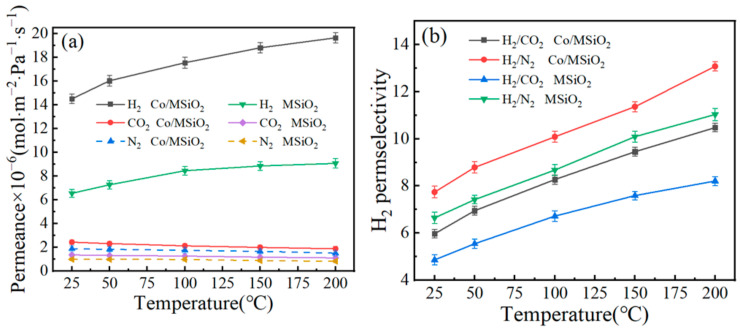
Influence of temperature on the (**a**) gases’ (H_2_, CO_2_, and N_2_) permeances and (**b**) H_2_ perm-selectivities for supported MSiO_2_ membrane and supported Co/MSiO_2_ membrane with *n*_Co_ = 0.08 at 0.2 MPa.

The temperature dependence of the various gases’ (H_2_, CO_2_, and N_2_) permeances and H_2_/CO_2_ perm-selectivities in the MSiO_2_ membrane and Co/MSiO_2_ membrane with *n*_Co_ = 0.08 at a pressure difference of 0.2 MPa are further investigated in the temperature range of 25–200 °C, which is depicted in Figure 9. In Figure 9a, with the continuous increase of temperature, the H_2_ permeance in MSiO_2_ and Co/MSiO_2_ membranes gradually increases, indicating that the permeation behavior of H_2_ in the two membranes mainly follows the activation-diffusion mechanism. In contrast, the permeances of CO_2_ and N_2_ are slightly decreased in a similar tendency to Knudsen diffusion in which molecules collide with pore walls more regularly than permeating molecules. In the case of activated diffusion, molecules permeate via micropores whilst being uncovered to repelling forces from the pore walls, and molecules with sufficient kinetic energy to conquer the repulsive force can permeate into the pores [38]. The decreasing permeances of CO_2_ and N_2_ are attributed to the violent movement of molecules and the increase of the mean free path when the temperature increases. As shown in Figure 9b, as temperature continues to increase in this range, the H_2_/CO_2_ and H_2_/N_2_ perm-selectivities for MSiO_2_ and Co/MSiO_2_ membranes all show a gradual, increasing trend. Compared with MSiO_2_ membrane, when operated at 25 °C, the permeance of H_2_, and the perm-selectivities of H_2_/CO_2_ and H_2_/N_2_ for Co/MSiO_2_ membrane increase by 121.53%, 22.76%, and 16.50%, respectively; on the other hand, when operated at 200 °C, those increase by 116.73%, 27.66%, and 18.53%, respectively. In addition, it can be found that, in the temperature range of 25–200 °C, the perm-selectivities of H_2_/CO_2_ and H_2_/N_2_ in both membranes are greater than the ideal perm-selectivities of Knudsen diffusion (4.69 and 3.74). The above results show that the Co/MSiO_2_ membrane has better perm-selectivity and permeance of H_2_ than those of MSiO_2_ membrane under same conditions.

The apparent activation energy (*E*_a_) is an index of the probability of molecules passing through shrinkage, so lower activation energy is related to higher permeability. According to the Arrhenius equation [16,38], permeability *F* is a temperature-related parameter, which can be expressed by modified Fick’s law:

(2) $$F=\exp(-\frac{E_{a}}{RT})$$

where *F* is the gas permeability, *E*_a_ is the apparent permeation-activation energy, *F*_0_ is a temperature-independent parameter, *R* is the constant of gas and *T* is the temperature of gas, and the unit is K. Equation (2) can be described in another form:

(3) $${\ln F=\ln F}_{0}-\frac{E_{a}}{RT}$$

**Figure 10:**
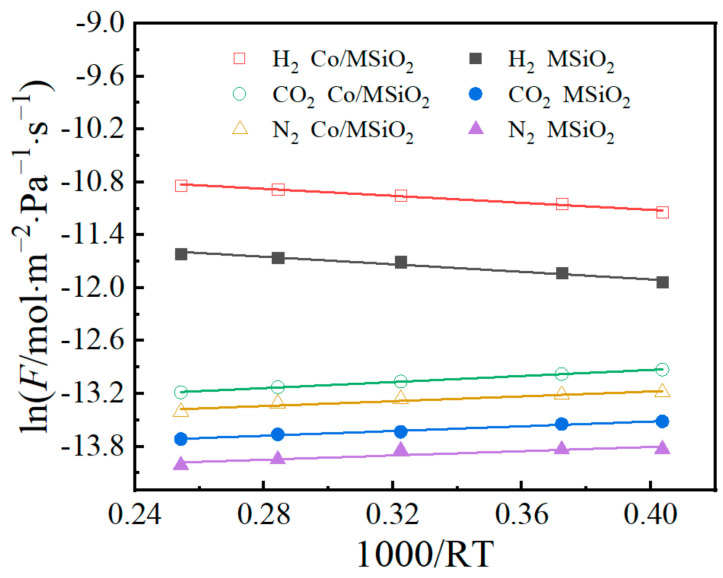
Arrhenius plots of gases’ (H_2_, CO_2_, and N_2_) permeances in supported MSiO_2_ membrane and supported Co/MSiO_2_ membrane with *n*_Co_ = 0.08 at 0.2 MPa.

In order to further study the diffusion phenomenon of gas molecules through MSiO_2_ and Co/MSiO_2_ membrane, the Arrhenius diagram is established, and the *E*_a_ values of gases’ (H_2_, CO_2_, and N_2_) permeations in MSiO_2_ and Co/MSiO_2_ film are calculated by using Arrhenius relationship between natural logarithm of permeation and reciprocal of temperature; the corresponding results are plotted in Figure 10. The *E*_a_ values of gases (H_2_, CO_2_, and N_2_) can be calculated from the Arrhenius formula for MSiO_2_ membrane and Co/MSiO_2_ membrane with *n*_Co_ = 0.08 at 0.2 MPa, which are listed in Table 2. It can be observed from Table 2 that the *E*_a_ value of H_2_ is positive, while the *E*_a_ values of CO_2_ and N_2_ are negative, which are conclusions that resemble those of previous reports [50]. The positive or negative value of *E*_a_ is related to the activated transportation behavior. The activation energy indicates the repulsive energy of osmotic substance passing through the pore structure of membrane [51]. Gas transport in the microporous state is carried out by heat-activated surface-diffusion mechanism [27]. The negative values *E*_a_ of CO_2_ and N_2_ indicate that there is a percolation path in the membrane, which is ample enough to enable the diffusion of larger molecules. The *E*_a_ values of gases (H_2_, CO_2_, and N_2_) in the Co/MSiO_2_ membrane are less than those in the MSiO_2_ membrane, which indicates that the structure of the Co/MSiO_2_ membrane is more open than that of the MSiO_2_ membrane, and the above observations are very consistent with the results of N_2_ adsorption-desorption. This result also suggests that the doping of cobalt successfully reduces the densification of SiO_2_ network. The larger porosity of Co/MSiO_2_ membrane leads to the kinetic energy of gas molecules overcoming the membrane pore barrier since it is less than that of MSiO_2_ membrane. Therefore, the gases’ (H_2_, CO_2_, and N_2_) permeances of Co/MSiO_2_ membrane are greater than those of MSiO_2_ membrane.

Table 3 shows perm-selectivities of H_2_, permeances of H_2_, *E*_a_ values of H_2_, and pore diameters for various SiO_2_ membranes prepared by other researchers using sol-gel process. As can be viewed from Table 3, it is hard to enhance the perm-selectivity and permeance of gas for the SiO_2_ membranes at the same time. Generally, larger average pore diameter leads to higher permeance of H_2_, lower perm-selectivity of H_2_, and smaller *E*_a_ value of H_2_. Besides, the *E*_a_ value of H_2_ has a link with the interplay between the molecules of H_2_ and the pore walls of the membrane. Therefore, probably due to the fact that the average pore diameters of the prepared Co/MSiO_2_ membranes are larger than that of the membrane obtained by different researchers listed in Table 3, it leads to smaller *E*_a_ values of H_2_ and higher permeance of H_2_. Due to the drawbacks for the conditions of experiment and technology, the perm-selectivities of H_2_ for the prepared Co/MSiO_2_ membrane is insufficient to reach significant value.

**Figure 11:**
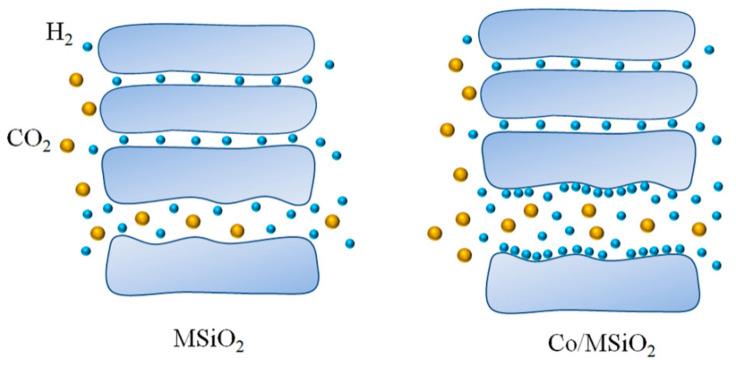
A possible schematic diagram for the change mechanism of supported MSiO_2_ and Co/MSiO_2_ membranes for separating H_2_/CO_2_.

The transport of H_2_, CO_2_, and N_2_ in MSiO_2_ membrane is controlled by molecular sieving, but Knudsen diffusion still exists due to the presence of small mesopores. However, thanks to the absorption for molecules of H_2_ by cobalt, the introduction of cobalt particles improves the surface diffusion of molecules of H_2_ in SiO_2_ membranes, which promotes the transmission of H_2_ and leads to the growth for permeance of H_2_ in the Co/MSiO_2_ membrane. Hence, we can find that, compared with CO_2_ and N_2_, the Co/MSiO_2_ membrane has the higher permeation rate to H_2_. Consequently, compared with MSiO_2_ membrane, the permeance of H_2_ and the perm-selectivities of H_2_/CO_2_ and H_2_/N_2_ in Co/MSiO_2_ membrane increase simultaneously. The possible change mechanism of MSiO_2_ and Co/MSiO_2_ membranes for separating H_2_/CO_2_ is schematically illustrated in Figure 11.

**Figure 12:**
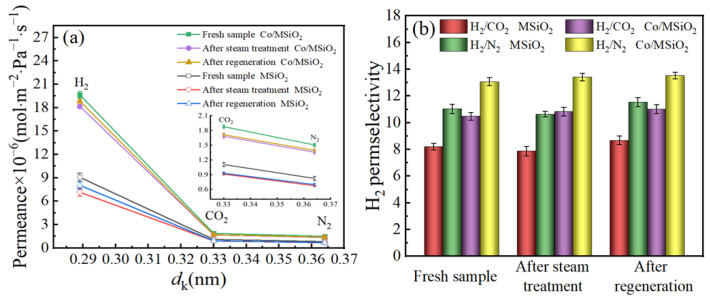
Effect of hydrothermal conditions on the (**a**) permeance of H_2_ and (**b**) perm-selectivities of H_2_ for supported MSiO_2_ membrane and supported Co/MSiO_2_ membrane with *n*_Co_ = 0.08 at a pressure difference of 0.2 MPa and 200 °C.

In order to look into the stability for MSiO_2_ membrane and Co/MSiO_2_ membrane with *n*_Co_ = 0.08 under hydrothermal conditions, they were subjected to saturated steam at 200 °C for 10 days and then regenerated by calcination at 350 °C. Figure 12 compares the effects of hydrothermal conditions on the both membrane samples. The experimental data were obtained at a pressure difference of 0.2 MPa and 200 °C. After steam treatment, the permeances of H_2_, CO_2_, and N_2_ for MSiO_2_ and Co/MSiO_2_ membranes appear to decrease. Compared with the untreated fresh samples, the permeance of H_2_ for MSiO_2_ and Co/MSiO_2_ membranes after steam aging for 10 days decrease by 21.06% and 7.48%, respectively, and the perm-selectivities of H_2_/CO_2_ and H_2_/N_2_ for MSiO_2_ membrane decrease by 4.13% and 3.54%, respectively, whereas those of Co/MSiO_2_ membrane increase by 3.37% and 2.55%, respectively. After regeneration by calcination at 350 °C, the permeances of gases (H_2_, CO_2_, and N_2_), the perm-selectivities H_2_/CO_2_ and H_2_/N_2_ for two membranes show an upward trend. However, compared with those of the untreated fresh samples, the permeances of H_2_ for MSiO_2_ and Co/MSiO_2_ membranes after regeneration decrease by 11.25% and 4.15%, respectively, whereas the perm-selectivities of H_2_/CO_2_ and H_2_/N_2_ for MSiO_2_ membrane increase by 5.80% and 4.64%, respectively, and those for Co/MSiO_2_ membrane increase by 5.03% and 3.49%, respectively. These results mightily indicate that regeneration causes structural changes of SiO_2_ membrane. The reduction of permeation for two membranes indicates that pore shrinkage of the membrane occurs after regeneration by calcination at 350 °C. The obtained smaller pores result in a lower permeance and higher perm-selectivity. However, a smaller decrease in permeation of H_2_ for Co/MSiO_2_ membrane indicates that the diffusion of hydrogen through the surface of cobalt particles could dominate permeation of H_2_. Therefore, the above results indicate that the Co/MSiO_2_ membrane has better hydrothermal stability and reproducibility than MSiO_2_ membrane.

### 3.6. SEM Analysis

**Figure 13:**
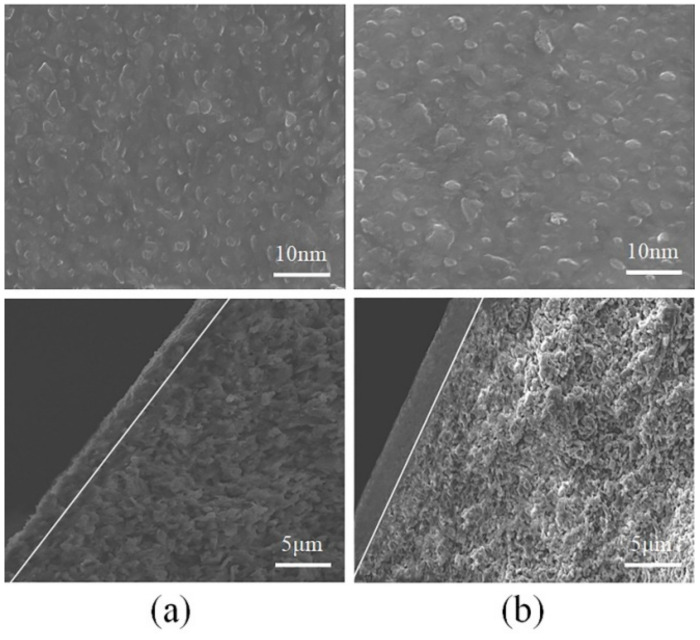
SEM images of membrane surface (top images) and cross-sections (bottom images) for supported Co/MSiO_2_ membranes with *n*_Co_ = (**a**) 0 and (**b**) 0.08 calcined at 400 °C under N_2_ atmosphere.

The SEM images of membrane surface and cross-sections for Co/MSiO_2_ membranes with *n*_Co_ = 0 and 0.08 calcined at 400 °C under N_2_ atmosphere are shown in Figure 13. In Figure 13a,b, it can be observed that there are no visible cracks and pinholes on Co/MSiO_2_ membranes surface, indicating that all membranes are well coated. Moreover, the particles on the surfaces of Co/MSiO_2_ membranes with *n*_Co_ = 0 are relatively uniform with the particle diameters in the range of 1.2–5.0 nm, while particle diameters of Co/MSiO_2_ membrane with *n*_Co_ = 0.08 are in the range of 1.6–6.3 nm. The cross-section of membrane indicates a classic, uneven configuration, which is related to the morphology of the supported SiO_2_ membrane. In the cross-section of the SEM image, there is a clear boundary between the support layer and selective layer. The selective layer is found to be smaller for Co/MSiO_2_ membrane with *n*_Co_ = 0, with a total thickness about 2.3 μm, whereas the wider selective layer with a total thickness of approximately 2.5 μm can be observed for Co/MSiO_2_ membrane with *n*_Co_ = 0.08. In addition, the consequences of gas-permeation measurements show that a complete selective layer has been successfully loaded on the support.

## 4. Conclusions

In summary, Co/MSiO_2_ materials and membranes with various *n*_Co_ were successfully synthesized under N_2_ atmosphere by sol-gel technique. The effect of *n*_Co_ on the microstructures and perm-selectivities of H_2_ for Co/MSiO_2_ membranes were investigated extensively. The results indicate that the cobalt element in Co/MSiO_2_ material calcined at 400 °C mainly exists in the form of Si–O–Co bond, Co_3_O_4_, and CoO crystals. The *n*_Co_ has little influence on the thermal stability of Si–CH_3_ groups of the methyl-modified silica materials. In addition, the introduction of metallic cobalt can enlarge the total pore volume and average pore diameter of the MSiO_2_ membranes. However, the *n*_Co_ has a large impact on the gas separation of Co/MSiO_2_ membrane. When operated at a pressure difference of 0.2 MPa and 200 °C, the Co/MSiO_2_ membrane with *n*_Co_ = 0.08 has better gas permeability and selectivity. Compared with MSiO_2_ membrane, the H_2_, CO_2_, and N_2_ permeances of Co/MSiO_2_ membrane with *n*_Co_ = 0.08 increased by 1.17, 0.70, and 0.83 times, respectively, and the perm-selectivities of H_2_/CO_2_ and H_2_/N_2_ increased by 27.66% and 18.53%, respectively. The *E*_a_ values of gases (H_2_, CO_2_, and N_2_) in the Co/MSiO_2_ membrane are less than those in the MSiO_2_ membrane. After steam treatment, the H_2_ permeance for MSiO_2_ and Co/MSiO_2_ membranes decreased by 21.06% and 7.48%, respectively, and the perm-selectivities of H_2_/CO_2_ and H_2_/N_2_ for MSiO_2_ membrane decreased by 4.13% and 3.54%, respectively, whereas those of Co/MSiO_2_ membrane increased by 3.37% and 2.55%, respectively. It is observed that, after regeneration, the permeances of gases (H_2_, CO_2_, and N_2_), the perm-selectivities H_2_/CO_2_ and H_2_/N_2_ for two membranes show an upward trend. However, compared with the untreated fresh samples, the permeances of H_2_ for MSiO_2_ and Co/MSiO_2_ membranes decreased by 11.25% and 4.15%, respectively, whereas the perm-selectivities of H_2_/CO_2_ and H_2_/N_2_ for MSiO_2_ membrane increased by 5.80% and 4.64%, respectively, and those for Co/MSiO_2_ membrane increased by 5.03% and 3.49%, respectively. The Co/MSiO_2_ membrane has better hydrothermal stability and reproducibility than MSiO_2_ membrane. In the future, we will further study these separation properties of Co/MSiO_2_ membranes for mixed gas with water vapor and compare the similarities and differences between the separation of single gas and mixed gas.

## Figures and Tables

**Table 1:** Parameters of pore structure for unsupported Co/MSiO_2_ materials with various *n*_Co_ after calcination at 400 °C.

Membrane Materials	BET Surface Area (m^2^/g)	Total Pore Volume (cm^3^/g)	Micropore Volume (cm^3^/g)	V_M_/V_t_	Mean Pore Size (nm)
*n*_Co_ = 0	389.38	0.230	0.150	0.652	1.75
*n*_Co_ = 0.08	775.34	0.424	0.045	0.106	2.34
*n*_Co_ = 0.15	644.10	0.402	0.035	0.087	2.73
*n*_Co_ = 0.5	494.54	0.399	0.034	0.085	3.14

**Table 2:** *E*_a_ values of gases (H_2_, CO_2_, and N_2_) calculated from Arrhenius formula for supported MSiO_2_ membrane and supported Co/MSiO_2_ membrane with *n*_Co_ = 0.08 at 0.2 MPa.

Gases	*E*_a_/KJ·mol^−1^	
	MSiO_2_	Co/MSiO_2_
H_2_	2.14	1.98
CO_2_	−1.34	−1.72
N_2_	−1.21	−1.37

**Table 3:** Perm-selectivities of H_2_, permeances of H_2_, *E*_a_ value of H_2_, and pore diameter for various SiO_2_ membranes prepared by other researchers using sol-gel process.

Membrane Type	Temperature and Pressure	Permeance of H_2_ (mol·m^−2^·Pa^−1^·s^−1^)	Perm-Selectivities of H_2_		Calcination Atmosphere	*E*_a_ Value of H_2_ (KJ·mol^−1^)	Pore Diameter (nm)
			H_2_/CO_2_	H_2_/N_2_			
Si(400) [49]	200 °C, 1 bar	1.74 × 10^−6^	7.5	64.4	Air	8	0.38–0.55
SiO_2_ [52]	200 °C, 2 bar	4.62 × 10^−7^	3.7	10.5	N_2_	—	0.30–0.54
SiO_2_–ZrO_2_ [53]	500 °C, 100 KPa	2 × 10^−6^	15	190	Air	13	—
Pd/SiO_2_ [25]	200 °C, 0.3 MPa	7.26 × 10^−7^	4.3	14	H_2_, N_2_	—	~0.57
Nb/SiO_2_ [54]	200 °C, 2 bar	5.03 × 10^−7^	3.5	6.5	N_2_	—	~0.55
Co/SiO_2_ [50]	200 °C, 500 KPa	5 × 10^−8^	31.6	—	Air	13.8	—
Co/SiO_2_ *	200 °C, 0.2 MPa	1.97 × 10^−5^	10.48	13.08	N_2_	1.98	0.3–2.3

* In this work.

## Data Availability

Not applicable.
